# Supplementary material for: Empirical methods for controlling false positives and estimating confidence in ChIP-Seq peaks
Source: BMC Bioinformatics. 2008 Dec 5;9:523. doi: 10.1186/1471-2105-9-523 (PMC2628906; doi:10.1186/1471-2105-9-523)
Supplement: Additional File 1 — A variety of html documents from the USeq web site detailing the available applications, their best usage, output file type descriptions, command line menus, etc. [file 1471-2105-9-523-S1.zip › USeqUserGuides/JavaDoc.zip!/JavaDoc/cgar/beans/ProcessedDataBean.html]

ProcessedDataBean


|  |  |  |
| --- | --- | --- |
| **Overview** \| **Package** \| **Class** \| **Use** \| **Tree** \| **Deprecated** \| **Index** \| **Help** | |  |
| **PREV CLASS**   **NEXT CLASS** | **FRAMES**    **NO FRAMES**     **All Classes** |  |
| SUMMARY: NESTED \| FIELD \| CONSTR \| METHOD | DETAIL: FIELD \| CONSTR \| METHOD |  |


---


## cgar.beans Class ProcessedDataBean

```
java.lang.Object
  cgar.beans.ProcessedDataBean
```

---

``` public class ProcessedDataBean extends java.lang.Object ```

Pointer to processed data file, where appropriate keep data broken down by chromosome.

---

**Constructor Summary**

`ProcessedDataBean()`


| **Method Summary** | |
| --- | --- |
| `int` | `getId()` |
| `java.lang.String` | `getName()` |
| `int` | `getProcessedDataTypeId()` |
| `java.lang.String` | `getUri()` |
| `boolean` | `isDasCompatible()` |
| `void` | `setDasCompatible(boolean dasCompatible)` |
| `void` | `setId(int id)` |
| `void` | `setName(java.lang.String name)` |
| `void` | `setProcessedDataTypeId(int processedDataTypeId)` |
| `void` | `setUri(java.lang.String uri)` |

**Methods inherited from class java.lang.Object**

`equals, getClass, hashCode, notify, notifyAll, toString, wait, wait, wait`

**Constructor Detail**

### ProcessedDataBean

```
public ProcessedDataBean()
```


**Method Detail**

### getId

```
public int getId()
```

---


### setId

```
public void setId(int id)
```

---


### getName

```
public java.lang.String getName()
```

---


### setName

```
public void setName(java.lang.String name)
```

---


### getProcessedDataTypeId

```
public int getProcessedDataTypeId()
```

---


### setProcessedDataTypeId

```
public void setProcessedDataTypeId(int processedDataTypeId)
```

---


### getUri

```
public java.lang.String getUri()
```

---


### setUri

```
public void setUri(java.lang.String uri)
```

---


### isDasCompatible

```
public boolean isDasCompatible()
```

---


### setDasCompatible

```
public void setDasCompatible(boolean dasCompatible)
```


---


|  |  |  |
| --- | --- | --- |
| **Overview** \| **Package** \| **Class** \| **Use** \| **Tree** \| **Deprecated** \| **Index** \| **Help** | |  |
| **PREV CLASS**   **NEXT CLASS** | **FRAMES**    **NO FRAMES**     **All Classes** |  |
| SUMMARY: NESTED \| FIELD \| CONSTR \| METHOD | DETAIL: FIELD \| CONSTR \| METHOD |  |


---
